# Clinical and patient-reported outcomes of self-administered outpatient parenteral antimicrobial treatment (S-OPAT): a scoping review

**DOI:** 10.1007/s15010-025-02549-1

**Published:** 2025-05-12

**Authors:** Jolanda M. Maaskant, Tessa de Vries, Laura Volle, Faridi S. Jamaludin, Suzanne E. Geerlings, Kim C. E. Sigaloff

**Affiliations:** 1 Department of Internal Medicine, Amsterdam UMC, Amsterdam, The Netherlands; 2 Amsterdam Public Health, Amsterdam, The Netherlands; 3 Department of Internal Medicine, Division of Infectious Diseases, Amsterdam UMC, Amsterdam, The Netherlands; 4 Biomedical Sciences, University of Amsterdam, Amsterdam, The Netherlands; 5 Research Support, Medical Library, Amsterdam UMC, University of Amsterdam, Amsterdam, The Netherlands; 6 Amsterdam Institute for Infection and Immunity, Amsterdam, The Netherlands; 7 Amsterdam University Medical Center, Meibergdreef 9, Amsterdam, 1105 AZ The Netherlands

**Keywords:** S-OPAT, Self-administration, Safety, Clinical outcome, Patient-reported outcome, Scoping review

## Abstract

**Purpose:**

This study aimed to provide a comprehensive overview of the existing literature on Self-administered Outpatient Parenteral Antimicrobial Therapy (S-OPAT), focusing on safety and clinical outcomes, factors influencing these outcomes, and the experiences of patients and caregivers.

**Methods:**

We searched the databases MEDLINE, CINAHL, Embase and Cochrane library. Publications were included if they reported on the clinical outcomes, safety, and/or experiences of patients and caregivers with S-OPAT. Study selection and data extraction were performed independently by two reviewers. Quantitative and qualitative data were summarized in data charting forms.

**Results:**

Forty-four studies were included: 41 primary studies, 2 systematic reviews and 1 clinical guideline. Clinical outcomes were reported in 17 and safety in 23 primary studies. Eleven studies compared S-OPAT to other OPAT delivery models. These studies showed that all models were generally comparable regarding clinical outcomes, but two studies reported an increased number of adverse events with S-OPAT. Nine studies, exploring a total of 7 potential risk factors, identified older age, comorbidities and *Staphylococcus aureus* infections as contributors to adverse events. The results of 14 studies on patient-centred outcomes showed that patients and caregivers considered S-OPAT a suitable alternative to other OPAT delivery models.

**Conclusion:**

We conclude that S-OPAT is a viable model of care, demonstrating favourable clinical outcomes, although some safety concerns have been reported. The growing care demand now and in the future urges further development of S-OPAT care. Gaps of knowledge still exist, and we provide recommendations for future research.

## Introduction

Outpatient parenteral antimicrobial therapy (OPAT), as defined by the Infectious Diseases Society of America, is ‘the administration of at least two doses of parenteral microbial therapy on different days without intervening hospitalization’ [1]. This approach allows patients who require parenteral antibiotic treatment to be discharged from the hospital, provided they are stable enough to continue their care at home. Since its inception in the 1970s, OPAT has been increasingly employed to facilitate medical treatment in a familiar environment, increase patient autonomy, reduce costs, increase hospital bed availability, and reduce the risk of nosocomial infections [2, 3]. A recent systematic review shows that OPAT is a safe and effective alternative to inpatient treatment [4].

Self-administered OPAT (S-OPAT) is a variant of OPAT in which parenteral antimicrobial treatment is administered by patients themselves, family members, or other caregivers, typically in the home setting. The term was coined in 2007 to distinguish this model from OPAT administered by healthcare [5]. However, the concept dates back further, as the first clinical report on OPAT already describes self-administration of treatment [6]. The advantage of S-OPAT is that it does not require dedicated mobile healthcare professionals for administration visits. Ideally, S-OPAT reduces healthcare staffing demands and costs, provides patients with greater control over their schedules, and enables the extramural use of antimicrobials administered in multiple doses per day, all while maintaining safety and effectiveness of treatment [2, 5].

A survey performed among experts from 28 European countries concluded that S-OPAT is offered in 10 countries but is considered standard practice only in Switzerland and the United Kingdom [7]. Wider use of S-OPAT may be hampered by uncertainty on its clinical outcomes and safety compared to other OPAT administration models. Moreover, there is a need for further understanding of which patients are best suited for S-OPAT, the factors that influence treatment outcomes, and the experiences of patients and their caregivers [8].

In this context, the aim of this scoping review is to provide a comprehensive overview of the existing literature on S-OPAT, focusing on clinical outcomes and safety of S-OPAT, factors influencing these outcomes, and the experiences of patients and caregivers. In addition, we aimed to identify gaps in knowledge and future directions of research.

## Methods

We performed a scoping review guided by the Joanna Briggs Institute methodology for scoping reviews [9] and reported according to the PRISMA extension for scoping reviews recommendations (PRISMA-ScR) [10].

### Search strategy

The search strategy was developed in consultation with a medical librarian of Amsterdam University Medical Centre (FJ). Search terms were based on the key components of S-OPAT: self-administration, outpatient setting, parenteral/intravenous administration, and antimicrobial agents. We searched the following online databases: MEDLINE, CINAHL, Embase and Cochrane library. As the term S-OPAT was first established in 2007, the search was limited to the period between 2007 and the search date (21 March 2024). We applied no restrictions based on language or geographical origin. The final search strategy is presented in Appendix A.

### Eligibility criteria

Considered for inclusion were publications providing information on the clinical outcome and safety of parenteral antimicrobial treatment administered by patients or their caregivers in an outpatient setting, or on the experiences of patients and caregivers involved in S-OPAT administration. We excluded publications that studied different OPAT delivery models but did not report results on S-OPAT separately. Participants of interest were any patients, including children, undergoing S-OPAT. Caregivers were considered participants of interest only in the context of their experiences with aiding with or administering the antimicrobial treatment. Primary research studies (quantitative, qualitative, and mixed methods), quality improvement reports, (systematic) reviews and guidelines were eligible for inclusion. We excluded editorials, letters to the editor, publications reporting the results of the same study, case studies, and guidance documents.

### Study selection

After removal of the duplicates, titles and abstracts were screened by two reviewers independently (LV, JMM). Disagreements were resolved by discussion and in consultation with a third reviewer (KS). In case of persistent uncertainty, a publication was passed onto the full text stage. All publications that were considered potentially relevant were screened full text by one reviewer (LV), with regular team discussions throughout the process. The reference lists of all included publications were hand-searched to seek for additional relevant publications. When full text was not available, corresponding authors were contacted. Rayyan Systems Inc. was used for managing the selection process. The literature selection process and results are visualized in a PRISMA flow chart.

### Data charting and synthesis of results

A data extraction form was constructed and piloted on 5 randomly selected publications and refined throughout this process. Data of the remaining publications were extracted by two reviewers (JMM, TV) independently. We resolved disagreements by consensus and discussion with the research team. After several discussion rounds, all researchers agreed upon the final version of the charting tables.

We extracted characteristics of the study, i.e. authors, year and country of publication, and study type. Furthermore, we collected data describing the population, the antimicrobial treatment, and the outcomes. The outcomes were divided into clinical outcomes (e.g. success and failure of the treatment) and safety (e.g. complications, adve**r**se events, hospitalizations, mortality) as defined in the publications. In addition, we extracted factors reported to impact the clinical outcomes and safety. Experiences of patients and caregivers were looked for both quantitatively and qualitatively. No quality assessment was executed, because scoping reviews are conducted to provide an overview of the existing evidence regardless of methodological quality or risk of bias [10, 11].

We summarized findings of the included studies by the types of outcomes narratively as well as in tabular form. If publications report on different OPAT delivery models, we only considered the S-OPAT data. Qualitative data on the outcome of interest were summarized, and descriptive statistics were used to present the quantitative data.

## Results

**Fig. 1:**
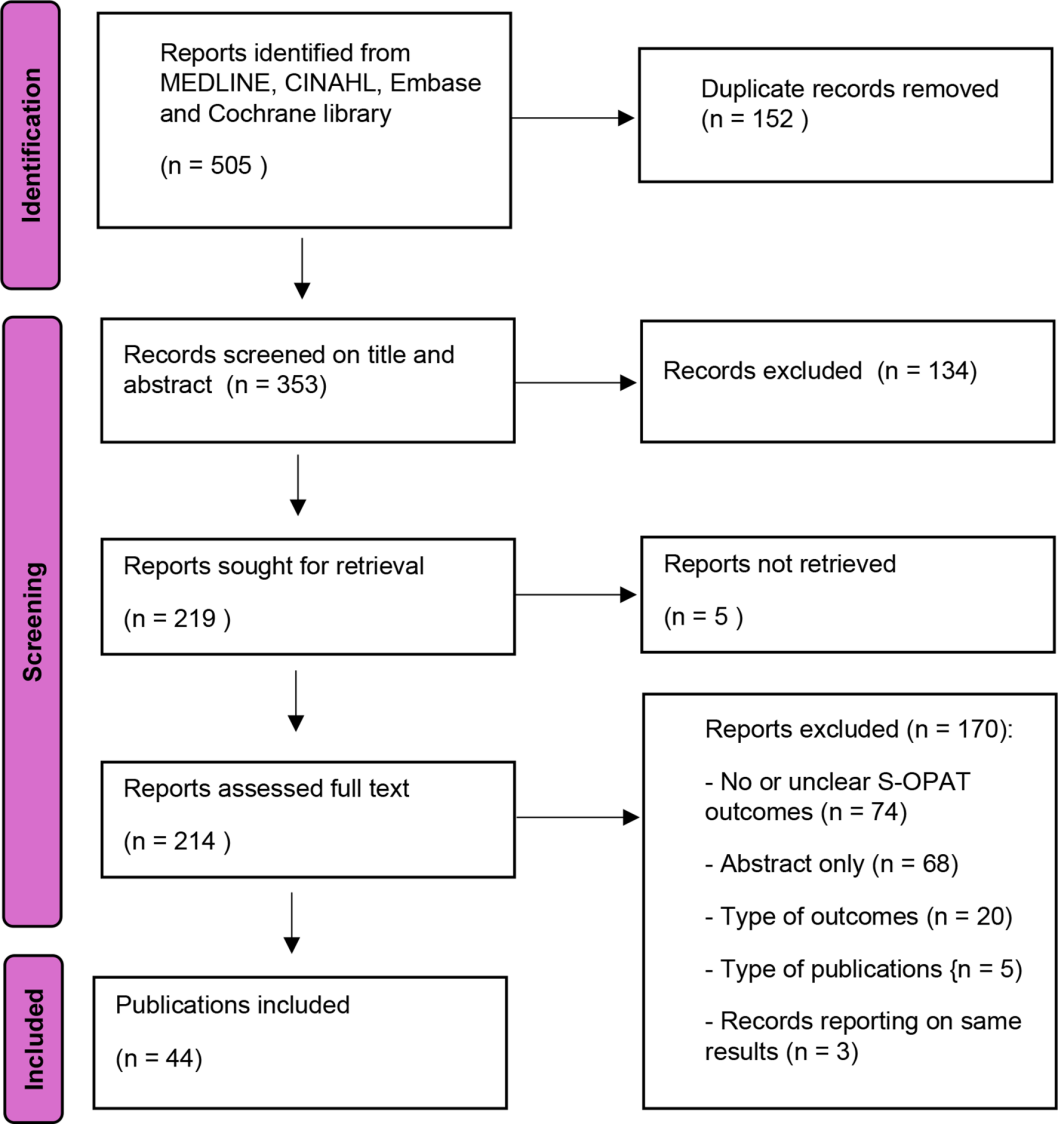
Selection process

After deduplication, the literature search yielded 353 potentially relevant publications, 219 of which were selected for full-text screening. The full text of 5 publications could not be retrieved. We excluded 170 publications based on the full-text screening, resulting in 44 studies for final inclusion [1, 5, 8, 12–52]. Cross-checking the reference lists did not yield new publications. The selection process is visualized in Fig. 1.

### Characteristics of the included studies

The characteristics of the 44 included studies are presented in Table 1.

Publications from 9 countries were included. Fifteen publications originated from the United States of America [1, 12, 13, 18, 23, 26–31, 35, 36, 38, 51], 10 from the United Kingdom [5, 8, 15–17, 20, 21, 25, 49, 50] and 8 from Spain [14, 19, 37, 39–42, 44]. Other publications originated from Germany [22, 33, 34, 45], Australia [47, 48], Switzerland [24, 43], Ireland [32], Singapore [46], and the Kingdom of Saudi Arabia [52].

Two systematic reviews [8, 14], one guideline [1], and 41 publications reporting original data were included [5, 12, 13, 15–52]. In total we found 35 cohort studies; 22 were retrospective [5, 12, 15–20, 23, 25, 26, 32–34, 36, 38, 42, 44, 47, 48, 50, 51], 12 prospective [13, 21, 22, 24, 27, 35, 37, 39, 40, 45, 46, 52], and 1 study used a combination [41].

Eleven studies compared S-OPAT with OPAT [5, 15, 16, 20, 25, 32, 42, 44, 46, 47, 50], 1 study compared S-OPAT with oral antibiotic therapy [35], and 9 studies explored factors influencing the clinical or safety outcomes of S-OPAT [13, 18, 23, 27, 36, 38, 40, 48, 51]. Fourteen studies described the findings in a single cohort [12, 17, 19, 21, 22, 24, 26, 33, 34, 37, 39, 41, 45, 52]. Other studies used qualitative study designs [28–30, 49] or surveys [17, 31–34, 37, 43, 51].

Three publications reported results of a paediatric population [12, 35, 47]. Thirteen publications focused on specific patient groups, i.e. non-injecting drug users [13], uninsured patients [16, 23], patients with periprosthetic joint infections [22], spinal infections [33], cranial infections [34], chronic respiratory diseases [14, 37, 39, 42], musculoskeletal infections [5, 51], and endocarditis [41]. Caregivers of adult patients participated in two studies [21, 29], and caregivers of pediatric patients in one [35].

**Table 1:** Characteristics of included studies

**Aggregated publications**							
**Author, date**	**Country**	**Type of publication**	**No of studies included**	**Type of studies included**		**Population**	
Balaguer, 2015	Spain	Systematic review	1	Randomized controlled trial		Adults and children with cystic fibrosis receiving intravenous antimicrobial treatment at home or in hospital	
Mitchell, 2017	UK	Systematic review	Total 128 S-OPAT 66	All studies, except case reports, reporting on OPAT and S-OPAT		Adult patients treated for any condition (and/or their caregivers), or practitioners involved in the delivery of OPAT	
Norris, 2018	USA	Clinical guideline	NA	NA		Adults and children treated for any condition	
**Primary studies**							
**Study or publication**			**Population**			**Antimicrobial treatment**	
**Author**,** date**	**Country**	**Type**	**Participants**	**Sample size**	**Indications** ^**1**^	**Antimicrobial agents** ^**2**^	**Duration**
Akar, 2014	USA	Retrospective chart review	Children, 53% with chronic underlying illnesses. Mean age 8.4 y.	Patients 183 Episodes 210	Musculoskeletal, bloodstream, pulmonary and intra-abdominal infections	Cefazolin, ceftriaxone, ceftazidim, clindamycin, ertapenem, vancomycin	Mean 16 d. (range 2–53)
Appa, 2020	USA	Prospective cohort study	Adult patients with and without non-injecting drug use. Median age 51 y. (IQR 40–60)	Patients 72: - Drug users 35 - No drug users 37	Bacteremia, osteomyelitis, abscess/complex soft tissue infection	NR	NR
Barr, 2012	UK	Retrospective cohort study	Adult patients. Mean age 55.1 y. (IQR 45.8–68.1)	Episodes 854	NR	NR	Median 41 d. (range 25.8–65.5)
Bhavan, 2015	UK	Retrospective cohort study	Uninsured patients. Range age 16 - ≥ 65 y.	Patients 1168: - S-OPAT 944 - OPAT 224	Infections bone and joint, skin and soft tissue, CNS, intra-abdominal, genitourinary, pulmonary, ENT, and bacteremia	NR	Median 26 d. at home
Bodycot, 2021	UK	Retrospective cohort study	Adult patients	Patients 958 Episodes 1084	Diabetic foot, urinary tract, and prosthetic knee-joint infections, osteomyelitis	NR	NR
Cox, 2007	USA	Retrospective cohort study	Adults patients. Total group mean age 58.8 y. (range 23–83). Group age *≥* 60 y. 68.9 y. (SD 8.9). Group age < 60 y. 50.6 y. (SD 8.1)	Patients 205 Episodes 231: - Age *≥* 60 y. 107 courses - Age < 60 y. 124 courses	Osteoarticular, skin and soft tissue infections, bacteremia	Cefazolin, ceftriaxone, ertapenem, vancomycin,	Group age *≥* 60 y. median 22 d. (range 2–107) Group age < 60 y. median 29.5 d. (range 4–450)
Dubois-Silva, 2023	Spain	Retrospective cohort study	Adult patients.	Episodes 31	NR	Meropenem	NR
Durojaiye, 2019	UK	Retrospective cohort study	Adult patients	Patients 105	NR	NR	NR
Eaves, 2014	UK	Prospective clinical evaluation	Adult patients, mean age 52 y. (range 21–79). Caregivers, mean age 63 y. (range 50–80)	Patients 29 Caregivers 9	Wide range, e.g. discitis, endocarditis, osteomyelitis and lung abscess	NR	Median 42 d. (range 28–168)
Frieler, 2021	Germany	Prospective cohort study	Adults patients. Mean age 66 y. (SD 15)	Patients 26 (23 with complete follow-up) Episodes 54	Periprosthetic joint infections and multidrug-resistant pathogens	Meropenem, vancomycin	Mean 66 d. (SD 26)
Ganguly, 2023	USA	Noninferiority retrospective cohort study	Uninsured adults patients. Mean age 50 y. (SD 13)	Patients 368: - Group Cefazolin 286 - Group Ceftriaxone 82	Methicillin-susceptible Staphylococcus aureus infection	Cefazolin, ceftriaxone	Mean 35 d. (SD 18)
Gardiol, 2016	Switzerland	Prospective cohort evaluation	Adult patients.	Patients 55	NR	NR	NR
Hatcher, 2019	UK	Retrospective analysis of prospectively collected data	Adult patients.	Episodes 1793: - S-OPAT 252 - OPAT 1541	NR	NR	NR
Karimaghael, 2021	USA	Retrospective cohort study	Adult patients with an elastomeric continuous infusion pump. Mean age 49.3 y. (SD 14.9)	Patients 91	Neurosyphylis, ocular/otosyphilis, osteomyelitis/septic arthritis	Nafcillin, penicillin	Mean 20.4 d. (SD 20.6)
Keller, 2018	USA	Prospective cohort study	Adult patient, median age 55 y. (IQR 41–63)	Patients 399	Bacteremia, abdominal infection, osteomyelitis, septic arthritis	Ceftriaxone, penicillins, piperacillin-tazobactam, vancomycin	Median 29 d. (IQR 15–44)
Keller, 2019	USA	Qualitative study	Adult patients. Interviews mean age 55 y. (SD 11.5). Observations mean age 51.5 y. (SD 15.2)	Patients 43 - Interviews 29 - Observations 14	NR	NR	NR
Keller, 2020 (a)	USA	Qualitative study	Adult patients and caregivers.	Patients 7 Caregivers 3	NR	NR	NR
Keller, 2020 (b)	USA	Qualitative study	Adult patients. Interviews mean age 55.4 y. (SD 12.5). Observations mean age 52 y. (SD 14.1)	Patients 60: - Interviews 40 - Observations 20	NR	NR	NR
Keller, 2022	USA	Survey	Adult patients. Mean age 57.5 y.	Patients 20	NR	NR	NR
Kieran, 2009	Ireland	Retrospective analysis of prospectively collected data	Adult patients.	Patients 56 Episodes 60: - S-OPAT 48 - OPAT 12	NR	NR	NR
Kilinc, 2023 (a)	Germany	Retrospective cohort study	Adult patients. Median age 52 y. (range 27–85)	Patients 52	Spinal infections, such as spondylodiscitis and spinal empyema	Cefazolin, ceftriaxone, fosfomycin, vancomycin,	Mean 41.3 d. (SD 16.4)
Kilinc, 2023 (b)	Germany	Retrospective cohort study	Adult patients. Median age 49 y. (range 20–71)	Patients 45	Cranial infections	Cefazolin, ceftriaxone, fosfomycin, meropenem, vancomycin	Mean 41.1 d. (SD 14)
Krah, 2018	USA	Prospective cohort study	Children and their caregivers	Patients and caregivers 89	Central nervous system, osteomyelitis and/or septic arthritis, endocarditis, pneumonia, intra-abdominal infection, musculoskeletal with hardware	Cefazolin, ceftriaxone, ertapenem	Antimicrobial use duration median 39 d. (IQR 24–95)
Lee, 2015	USA	Retrospective review	Adult patients. Mean age 58 (SD 17)	Patients 400	Osteoarticular infections, bacteremia, abscess	Cefazolin, ceftriaxone, ertapenem, oxacillin	NR
Lopez-Cortes, 2019	Spain	Prospective cohort study	Adult patients. Median age 71 y. (IQR 56–80)	Patients 67	Exacerbation of Non-Cystic Fibrosis Bronchiectasis	Ceftazidime, piperacillin/tazobactam, combination including meropenem, tobramycin,	Median 12 d. (IQR 9–13)
Matthew, 2007	UK	Retrospective cohort study	Adult patients. S-OPAT mean age 46.2 y. OPAT mean age 60.9 y.	Episodes 2482: - S-OPAT 473 - OPAT 2009	Musculoskeletal infections (both groups)	Ceftriaxone, teicoplanin	Mode 6 weeks both S-OPAT and OPAT
Mohammadi, 2013	USA	Retrospective chart review	Adult patients. Mean age 63.2 (range 31–87)	Patients 190	Osteomyelitis, urinary tract, skin and soft tissue, bloodstream, and prosthetic joint infection, endocarditis.	Ceftriaxone, nafcillin, vancomycin	Median 30 d. (range 5–56)
Monton, 2013	Spain	Prospective cohort study	Adult patients. Mean age 64 (SD 17)	Patients 50 Episodes 74	Exacerbation of a chronic respiratory disease	NR	Mean 19 d. (SD 13)
Mujal, 2016	Spain	Prospective cohort study	Adult patients. Group < 65 y. mean age 47.7 y. (SD 13.3). Group 65–79 y. mean age 72.3 y. (SD 4.1). Group *≥* 80 y. mean age 84.9 y. (SD 3.7)	Patients 420: - Group < 65 y. 139 - Group 65–79 y. 182 - Group *≥* 80 y. 99	Respiratory, urinary, intra-abdominal, osteoarticular, skin infections	Ceftriaxone, cefepime/ceftazidime, ertapenem, meropenem, piperacillin/tazobactam, vancomycin/teicoplanin	NR
Pajaron, 2017	Spain	Cohort study with retrospectively and prospectively collected data	Adults patient. Mean age 61 (SD 16.5)	Patients 54 Episodes 57	Infectious endocarditis	Ampicillin, ceftriaxone, cloxacillin, daptomycin, linezolid, penicillin G, teicoplanin, vancomycin	Mean 3.1 w. (SD 0.9)
Ponce Gonzalez, 2017	Spain	Retrospective cohort study	Adults patients.	Patients 174	Acute exacerbation of COPD	Amikacin, cefepime, ceftazidim, ceftriaxone, ertapenem, levofloxacin, meropenem, piperacillin/ tazobactam	NR
Saillen, 2017	Switzerland	Survey	Adult patient. Mean age 56 y. (SD 15)	Patients 71	Osteoarticular, urinary tract, endovascular, intra-abdominal, skin and soft-tissue infections, endocarditis	NR	Mean 19.2 d. (SD 16.6)
Sanroma, 2018	Spain	Retrospective study	Adult patients.	Patients 371	NR	Ertapenem	Median 9 d. (range 6–13)
Schmidt-Hellerau, 2024	Germany	Prospective observational study	Adult patients. Median age 56 y. (IQR 40–66)	Patients 77	Joint and bone infections, vertebral osteomyelitis, periprosthetic joint infection, septic arthritis, bacteremia, endocarditis, neurosyphylis	Ceftriaxone, flucloxacillin, Meropenem, penicillin, vancomycin	Median 15 d. (IQR 11–26, range 5–127)
Seetoh, 2013	Singapore	Prospective cohort study	Adult patients.	Episodes 397	NR	NR	NR
Sriskandarajah, 2020	Australia	Retrospective review	Pediatric patients. Mean age 9.13 (SD 5.12)	Episodes 100: - S-OPAT 49 - OPAT 51	Cystic fibrosis, osteomyelitis, pneumonia, septic arthritis, bacteremia, primary ciliary dyskinesia	Tobramycin in combination with ceftazidime or piperacillin/tazobactam	Median 14.5 d. (IQR 14.5 -24.75). OPAT median 11 d. (IQR 8–14)
Subedi, 2015	Australia	Retrospective analysis of prospectively collected data	Adult patients. Median age 55 (range 16–90)	Patients 144 Episodes 150	Osteomyelitis, septic arthritis, endocarditis, bacteremia, skin and soft-tissue, surgical site, central nervous system, and intra-abdominal infections	Benzylpenicillin, cefazolin, ceftazidime, ceftriaxone, clavulanic acid, ertapenem, flucloxacillin, lincomycin, meropenem, teicoplatin ticarcillin-vancomycin	Median 22 d. (range 4–106)
Twiddy, 2018	UK	Qualitative study	Adult patient.	Patients 5	NR	NR	NR
Underwood, 2019	UK	Retrospective analysis of prospectively collected data	Adult patients.	Episodes 30	NR	NR	NR
Yagnik, 2022	USA	Retrospective chart review	Adult patients. Mean age 49 y. (SD 13)	Episodes 200: - Drip infusion method 95 - Prefilled syringe delivery method 105	Bone and joint infection	Cefazolin, cefepime, ceftriaxone, daptomycin	NR
Zikri, 2021	SAU	Prospective cohort study	Adults patients. Mean age 53 y. (range 17–77)	Patients 47	Urinary tract, diabetic foot, respiratory tract, intra-abdominal and cardiovascular infections	Ceftriaxone, imipenem, meropenem, piperacillin/tazobactam	NR

CNS: Central Nervous System; COPD: Chronic Obstructive Pulmonary Disease; ENT: Ear/Nose/Throat; IQR: interquartile range; NA: not applicable; NR: not reported (for the S-OPAT participants); PICC: peripherally inserted central catheter; SD: standard deviation; SAU: Kingdom of Saudi Arabia; TCVC: tunneled central venous catheter; UK: United Kingdom; USA: United States of America; y: years; d: days; w: weeks

^1^ Most frequently reported indication; ^2^ Most frequently reported antimicrobial agents

### Aggregated publications of S-OPAT

Our search yielded two systematic reviews [8, 14] and one guideline [1]. The systematic review by Mitchell et al. included 128 studies that evaluated existing evidence on the efficacy, safety, acceptability, and cost-effectiveness of different OPAT delivery models. Studies that evaluated S-OPAT as a single model of delivery were few (16/128, 12.5%) and published before the inclusion period of our review [8]. The systematic review by Balaguer et al. included one randomized controlled trial evaluating S-OPAT versus hospital intravenous antibiotic treatment for cystic fibrosis [14]. This study originated from 1997 and was therefore excluded from our review. The 2018 IDSA guidelines strongly recommend patients to be allowed self-administration, based on low-quality evidence, but on vast clinical experience with S-OPAT [1].

### Outcomes reported in the primary studies

Clinical and safety outcomes of S-OPAT are presented in Table 2.

#### Clinical outcome

We found 17 publications reporting on clinical outcomes [12, 17, 22, 25, 26, 32–34, 37, 39, 41, 43–47, 52], of which 5 studies evaluated the clinical outcome of S-OPAT in comparison with OPAT care delivered by healthcare professionals, either in specialized OPAT clinics [46], at the patients’ homes [32, 42, 44], or a combination [25].

Comparison of S-OPAT with OPAT delivered by healthcare professionals at the patients’ homes, e.g. community nurses, showed no differences in clinical outcome [25, 32, 42, 44]. The study by Hatcher et al. also compared outcomes of S-OPAT patients with patients receiving care at specialized OPAT clinics. Their results showed that clinic-based OPAT was associated with an increased odds of OPAT success, defined as survival without readmission (OR 2.1, 95%CI 1.03–2.28, *p* = 0.02) [25]. However, Seetoh et al. reported no significant difference between S-OPAT and clinic-based OPAT on unplanned readmission or death [46].

#### Safety

We found 23 publications reporting on safety [5, 12, 15–17, 19, 20, 22, 24–26, 32–34, 37, 39, 41, 42, 44, 45, 47, 50, 52]. Nine studies evaluated the safety of S-OPAT in comparison with OPAT care delivered by healthcare professionals, either in specialized OPAT clinics [15, 20, 50], at the patients’ homes [5, 32, 42, 44, 47], or a combination [16].

Bhavan et al. reported a significant difference between S-OPAT and a healthcare professional OPAT model on 30-days all cause readmission in favor of S-OPAT (16.7% versus 23.7%, adjusted HR 0.53, 95%CI 0.35–0.81, *p* = 0.003) [16]. No significant differences were found between S-OPAT and other OPAT delivery models on catheter events [15, 32, 42], adverse drug events [32, 42], complications [5], infection-related complications [42], readmissions [5, 20, 32, 44], and mortality [16, 44].

Three publications evaluated S-OPAT as a potential risk factor for adverse safety outcomes [20, 47, 50]. S-OPAT was not associated with 30-days unplanned hospitalization [20]. However, S-OPAT was significantly associated with the risk for adverse events (adjusted OR 6.25, 95%CI 1.44–27.15) [47], and catheter-related adverse events (HR 4.15, 95%CI 1.7–9.1, *p* = 0.007) [50].

**Table 2:** Clinical outcomes and safety of S-OPAT

Author, date	Clinical outcomes		Safety	
	Definition	Results	Definitions	Results
Comparative studies				
Barr, 2012	NR	NR	- Line infection (local infection, bloodstream infection) - Other line events (chemical or mechanical phlebitis, leakage, extravasation, occlusion, non-electively removal)	S-OPAT versus OPAT (corrected for confounders): - Line infection 11/532 (2%) versus 9/298 (2.9%), NS - Other line events 91/452 (16.8%) versus 34/273 (11.1%), NS
Bhavan, 2015	NR	NR	- 30-days all cause readmission - 1-year all cause mortality	S-OPAT versus OPAT (corrected for confounders): - Readmission 158/944 (16.7%), versus 53/224 (23.7%), HR 0.53, *p* = 0.003 - Mortality 51/944 (5.4%) versus 10/244 (4.5%), NS
Durojaiye, 2019	NR	NR	30-days unplanned hospitalization	In a multivariable model, S-OPAT was not significantly associated with 30-days unplanned hospitalization, NS
Hatcher, 2019	S-OPAT success (no readmission due to infection worsening or due to adverse event, and no mortality by any cause during OPAT)	S-OPAT success 233/252 (92.5%) Multivariable analyses showed - OPAT-clinic versus S-OPAT: OR 2.1, 95%CI 1.03–2.28, *p* = 0.02. - OPAT-community nurse versus S-OPAT, NS	S-OPAT-related adverse events (drug-related, line-related)	S-OPAT-related adverse events 16/252 (6.3%)^1^
Kieran, 2009	Clinical cure	52/56 patients (93%) Differences between S-OPAT and OPAT, NS. No further information provided.	- Readmissions related to S-OPAT - AEs (line-related complications, drug-related adverse events	- Readmissions: 5/60 (8%) - Line-related complications: 5/60 (8%) - Drug-related adverse events: 4/60 (7%) Differences between S-OPAT and OPAT, NS. No further information provided.
Matthews, 2007	NR	NR	- Complication rate (side effects or intolerance of drugs, complications related to vascular access device) - Readmissions	S-OPAT versus OPAT: - Complication rate 112/473 (24%) versus 353/1536 (23%), NS - Readmissions 50/473 (10.5%) versus 193/1536 (10.5%), NS
Ponce Gonzalez, 2017	Effective treatment (absence of medical complications, absence of signs of acute disease, no ER visits, no readmissions, return to basal clinical situation)	93.4% Univariable analysis showed no differences between S-OPAT and OPAT	- Adverse drug events - Catheter-related complications - Infection-related complications	- Adverse drug events: 2.3% - Catheter-related complications: 2.7% - Infection-related complications: 5.7% Univariable analysis showed no differences between S-OPAT and OPAT
Sanroma, 2018	Cure or improvement (resolution or partial resolution of symptoms)	Multivariable analysis showed a non-significant association of S-OPAT with risk of treatment failure/relapse compared to OPAT	- S-OPAT related readmissions - Death	- Readmissions: 15/371 (4%) - Death: 13/371 (3.5%) Multivariable analysis showed a non-significant association of S-OPAT with safety outcomes compared to OPAT
Seetoh, 2013	Clinical deterioration (worsened co-morbidity or worsened infectious disease for which OPAT was initiated, leading to unplanned re-admission or death)	9.6% Multivariable analysis showed no differences between S-OPAT and OPAT, NS	NR	NR
Sriskandarajah, 2020	Completion of treatment as prescribed by pediatrician	S-OPAT: 86/100 (86%)^1^	- Unplanned readmission - AEs related to vascular access device - AEs related to infusion device - AEs related to antimicrobials	- Readmission: 8/100 (8%) - AEs vascular access device: 16/100 (16%) - AEs infusion device: 4/100 (4%) - AEs antimicrobials: 0 Adjusted for covariates, S-OPAT was significantly associated with adverse events versus OPAT: OR 6.25, 95%CI 1.44-27.15
Underwood, 2019	NR	NR	Catheter-related adverse events	- Adverse events: 8 (27%) or 12 (95%CI 6.0-23.9)/1000 catheter-days Adjusted Poison regression analysis showed S-OPAT was associated with a higher rate of catheter-related adverse events compared to OPAT: HR 4.15, 95%CI 1.7–9.1, *p* = 0.007
Descriptive studies				
Akar, 2014	Antibiotic– organism mismatch (antibiotic resistant against the isolated organism, use of an antibiotic with a too broad or too small spectrum)	22/210 (10.5%)	- Mechanical complication (dislodgement, displacement, thrombosis, damage to the catheter) - Antibiotic toxicity (neutropenia, hepatitis, colitis, rash) - Catheter-associated bloodstream infection - S-OPAT related ED visits or hospitalisations	- Mechanical complications: 21/210 (10%) - Antibiotic toxicity: 26/210 (12.4%) - Catheter-associated bloodstream infections: 3/210 (2.6%) - ED visits or hospitalisations: 20/210 (9.5%)
Bodycot, 2021	- Infection improved or cured. - S-OPAT process successfull or partially successful	- Infection improved or cured: range 84.6% − 92.8% - S-OPAT (partially) successful: range 75%- 91.4%	Serious vascular access device events (thrombus, catheter-related bloodstream infection)	- Thrombus: 11/1084 (1%) - Catheter-related bloodstream infection: 2/1084 (0.2%)
Dubois-Silva, 2023	NR	NR	- Catheter-related bloodstream infection - Readmission due to vascular access complication	- Catheter-related bloodstream infection: 0 - Readmission: 0
Frieler, 2021	Successful treatment (infection eradication, no subsequent surgical intervention after revision surgery, no infection-related mortality)	21/23 (91%)	- Adverse drug reactions - Catheter-related complications	- Adverse drug reactions: 1/26 (2%) - Catheter-related complications: 5/54 (9%)
Gardiol, 2016	NR	NR	- Catheter-related bloodstream infection (positive blood culture). - OPAT-related readmission - Mortality	- Catheter-related bloodstream infection: 1/55 (2%) - OPAT-related readmission: 2/55 (4%) - Mortality: 0
Karimaghael, 2021	- Treatment completion - Cure from infection	Treatment completion 83/91 (92.2%) Cure from infection 85/91 (93.4%)	- 30-days readmission related to S-OPAT - 30-days ED visits related to S-OPAT - ED visits during and related to S-OPAT - Central line-associated issues - Antibiotics-related side effects	- 30-days readmission: 1/91 (1.1%) - 30-days ED visits: 15/91 (16.5%) - ED visits during S-OPAT: 15/91 (16.5%) - Central line-associated issues: 13/91 (14.3%) - Antibiotics-related side effects: 6/91 (6.6%)
Kilinc, 2023 (a)	Treatment failure	1 (2%)	- Adverse effects or complications regarding laboratory parameters - PICC line complications - Readmission	- Adverse effects or complications: 0 - PICC line complications: 0 - Readmission: 1/52 (2%)
Kilinc, 2023 (b)	Treatment failure	3/45 (6.7%)	- Readmission	- Readmission 5/45 (11.1%)
Lopez-Cortes, 2019	Resolution of the exacerbation (resolution of fever, resolution or reduction of purulent secretions, improvement of dyspnea, normalization of acute-phase reactants, and improvement of oxygen saturation levels)	63/67 (94%)	- Readmission - Mortality - Nephrotoxicity - Catheter-associated bacteremia	- Readmission: 4/67 (6%) - Mortality: 1/67 (2%) - Nephrotoxicity: 2/67 (3%) - Catheter-associated bacteremia: 0
Monton, 2013	Satisfactory clinical outcome	44/50 (87%)	- Readmission during S-OPAT - Complications vascular access device	- Readmissions: 6/50 (13%) - Complications: (minor): 15/50 (30%)
Pajaron, 2017	Relapse of the infectious endocarditis	3 patients (5.2%)	- Mortality - Complications resolved home - Unexpected readmissions	- Mortality: 0 - Complications: 11/57 (19.2%) - Readmissions: 6/57 (10.5%)
Schmidt-Hellerau, 2024	Curation or switch to oral antibiotic treatment	70/74 (95%)	- Missed administrations more than 1 - All-cause readmissions - S-OPAT related readmission - Severe catheter-related AEs (CTCAE grade 3 and 4)	- Missed administrations: 4/77 (5%) - All-cause readmissions: 16/77 (21%) - S-OPAT related readmission: 1/77 (1%) - Catheter-related AEs: 4/77 (5%)
Zikri, 2021	Relaps of infection	6/47 (12.8%)	- ED visits S-OPAT related - Readmissions S-OPAT related - Mortality	- ED visits: 42/47 (89%) - Readmissions: 4/47 (8.5%) - Mortality: 0

AE: Adverse Event; CTCAE: Common Terminology for Adverse Events; ED: Emergency Department; HR: Hazard Ratio; OR: Odds Ratio; NR: not reported; NS: not statistically significant; 95%CI: 95% confidence interval; aIRR: adjusted incidence rate ratio

^1^ Results only descriptively reported

#### Factors impacting S-OPAT clinical outcome and safety

A summary of the factors impacting S-OPAT clinical outcome and safety as reported in the included studies is presented in Table 3. Seven potential risk factors were studied in 9 studies: age [18, 40], drug use [13], comorbidity [38, 48], microbiological diagnosis [27], antimicrobial agent [23, 27, 36], catheter type [27], and delivery method [51]. Patients with two or more comorbidities had an increased risk of treatment failure [48], and diabetes resulted in an increased risk of relapse at 90 days post treatment [38]. *Staphylococcus aureus* infections were associated with an increased rate of catheter complications [27]. Older age increased the risk of nephrotoxicity [18], and readmission rates due to worsening of underlying diseases [40]. Some studies looked at individual antimicrobial agents in detail and found that cefazolin, vancomycin and daptomycin were associated with more catheter complications [23, 27]. Lee et al. reported that antibiotic switches for adverse events were more frequent with oxacillin use [36]. Midline catheters were associated with an increased rate of catheter complications [27]. However, drip infusion or prefilled syringe delivery methods did not influence safety [51].

**Table 3:** Factors impacting S-OPAT clinical outcome and safety

Factor	Author, date	Clinical outcomes		Safety	
		Definition	Results	Definitions	Results
Age	Cox, 2007	Failure of treatment	- Group age *≥* 60 y. versus group age < 60 y.: 9 (8%) versus 7 (6%), NS	- Allergic reactions (rash, urticaria, anaphylaxis), nephrotoxicity, leucopenia, neutropenia, thrombocytopenia, eosinophilia - Venous access device complications (deep venous thrombosis, phlebitis, accidental removal of device, leaking, local infection and device-related bloodstream infection) - Hospital readmission - Urgent care visit related to S-OPAT	Results/1000 home iv days, group age *≥* 60 y. versus group age < 60 y. - Nephrotoxicity: 3.03 versus 0.46, *p* = 0.02 - Urgent visits: 31.4 versus 14.3, *p* < 0.001 - Other outcomes, NS
	Mujal, 2016	Inadequate control of infection leading to antibiotic switch, readmissions	Group < 60 y. versus Group 65–79 y. versus Group *≥* 80 y.: 29 (21%) versus 17 (9%) versus 18 (18%), NS	- Adverse drug events - Catheter-related complications - Readmission < 30 days after discharge all cause - Readmission due to worsening of underlying diseases	Group < 60 y. versus Group 65–79 y. versus Group *≥* 80 y. (corrected for confounders): - Adverse drug events: 7 (5%) versus 5 (2.7%) versus 3 (3%), NS - Catheter-related complications: 22 (15.8%) versus 32 (17.6%) versus 20 (20.2%), NS - Readmission < 30 days after discharge all cause: 27 (19.4%) versus 47 (25.8%) versus 27 (27.3%), NS - Readmission due to worsening of underlying diseases: 15 (10.8%) versus 38 (20.9%) versus 19 (19.2%), *p* = 0.05
Comorbidity	Mohammadi, 2013	Overall clinical cure at end of treatment and 90-days post S-OPAT	At end of treatment 148 (78%) 90-days post S-OPAT 110 (58%) After corrected for infection type, patients with diabetes had an increased risk of relapse at 90 days post S-OPAT: OR 1.76, 95%CI 1.57–196, *p* = 0.03	- AEs (neutropenia, diarrhea, rash, nephrotoxicity) - Complications related to vascular access device - Readmissions - ED visits	- AEs: 12 (6.3%) - Vascular access device complications: 5 (2.6%) - Readmissions 3 (1.6%) - ED visits 2 (1.1%)
	Subedi, 2015	Treatment success (cure or major improvement, decrease in C-reactive protein, no relapse within 28 days after end of treatment)	93% Multivariate analysis showed patients with 2 or more comorbidities had an increased risk of treatment failure: OR 2.15, 95%CI 1.28–3.65, *p* = 0.004	- Drug-related complications (e.g. rash, hepatitis, gastrointestinal symptoms) - Line-related complications (line infection, thrombosis, leakage, accidental removal) - Readmissions	- Drug-related: 11 (7%) - Line-related: 5 (3%) or 1.4/1000 catheter-days - Readmissions 9 (6%)
Drug use	Appa, 2020	Treatment completion	Drug use versus no drug use: 91% versus 89%, NS	- Hospital readmissions (30-days and 90-days)	Drug use versus no drug use: - 30-days 3 versus 0, NS - 90-days 3 versus 2, NS
Micro-biological diagnosis	Keller, 2018	NR	NR	- Catheter complications (occlusion, thrombosis, extravasation, phlebitis)	- Patients: 43 (12.7%) - 2.62/1000 OPAT days Factor associated with catheter complications: - Staphylococcus aureus infection (aIRR 2.13, 95%CI 1.09–4.19)
Antimicrobial agent	Ganguly, 2023	Treatment failure (repeat positive blood culture or retreatment within 6 months	Cefazolin versus Ceftriaxone: 10 (4%) versus 2 (2%), NS	- 30-days all-cause readmission rate - Central line-associated bloodstream infection	Cefazolin versus Ceftriaxone: - Readmission rate 62 (22%) versus 17 (21%), NS - Central line-associated bloodstream infection 31 (11%) versus 2 (2%), *p* = 0.02
	Keller, 2018	NR	NR	- Catheter complications (occlusion, thrombosis, extravasation, phlebitis)	Associated with catheter complications: - Daptomycin (aIRR 4.45, 95%CI 1.02–19.41) - Vancomycin (aIRR 2.32, 95%CI 1.20–4.46)
	Lee, 2015	Clinical cure (resolution of signs and symptoms of infection and discontinuation of antibiotic therapy)	Total 287 (72%) After correction for confounding: oxacillin showed a lower treatment success rate (HR 0.53, 95%CI 0.39–0.74) compared to ertapenem	- Adverse drug reaction (diarrhea, fever, transaminitis, neutropenia, rash, acute renal injury) - Outpatient antibiotic switch - Readmissions within 30 days	- Adverse drug reaction: 47 (11.6%) - Outpatient antibiotic switch: 50 (12.5%) - Readmissions: 67 (16.8%) After correction for confounding: oxacillin showed higher antibiotic switches due to adverse drug reactions or treatment failure (HR 5.06, 95%CI 1.74–14.75) compared to ertapenem.
Catheter type	Keller, 2018	NR	NR	- Catheter complications (occlusion, thrombosis, extravasation, phlebitis)	- Patients: 43 (12.7%) - 2.62/1000 OPAT days Factor associated with catheter complications: - Midlines (aIRR 9.44, 95%CI 1.12–41.97)
Delivery method	Yagnik, 2022	NR	NR	- All cause 30-days readmissions - All cause 1-year readmissions - ED visits within 30 days - ED visits within 1 year - Mortality	- 30-days readmissions: 21 (11%) - 1-year readmissions: 66 (33%) - ED visits 30 days: 46 (23%) - ED visits 1 year: 87 (44%) - Mortality: 11 (5%) There were no significant differences between the drip infusion and prefilled syringe delivery method.

AE: Adverse Event; ED: Emergency Department; HR: Hazard Ratio; OR: Odds Ratio; NS: not statistically significant; 95%CI: 95% confidence interval; aIRR: adjusted incidence rate ratio

#### Patient-centred outcomes

A summary of the patients-centred outcomes is presented in Table 4. We found 14 publications reporting on patient-centered outcomes [17, 21, 28–35, 37, 43, 49, 51]. Seven studies explored patient-centred outcomes as secondary research objectives [17, 32–35, 37, 51]. S-OPAT compared to OPAT showed more favorable outcomes for S-OPAT [43]. S-OPAT compared to oral antibiotics showed better patient-centred outcomes for oral antibiotics [35]. Over 90% of the participants preferred antibiotic treatment at home rather than in hospital [17, 32, 37], and satisfaction with S-OPAT was high [31–34, 43, 51]. At discharge and during the S-OPAT period patients and caregivers were comfortable with S-OPAT [31, 35], and the burden for caregivers of pediatric patients seemed acceptable [35]. Two studies report high levels of competency of patients and caregivers after S-OPAT training [21, 51]. Besides the positive results, barriers with S-OPAT, such as restricted mobility and infection prevention, are reported as well [28–30].

**Table 4:** Patients- and caregivers-centred outcomes

Author, date	Participants	Sample size	Data collection	Results
Comparative studies				
Krah, 2018	Pediatric patients and their caregivers	89	- Self-constructed survey exploring comfort with the S-OPAT process and burden of administration (5-point Likert scale) - Family impact module Paediatric Quality of Life Inventory	Comparison between oral treatment versus S-OPAT: - Comfort with the treatment at discharge: mean 2.87 (SD 1.51) versus 2.09 (SD 1.46), *p* < 0.001 - Comfort with the treatment at follow-up: mean 3.02 (SD 1.59) versus 2.82 (SD 1.61), NS - Burden of drug administration caregiver: mean 0.64 (SD 0.84) versus 1.30 (SD 1.11), *p* < 0.001 - Burden of drug administration patient: mean 1.04 (SD 1.15) versus 1.38 (SD 1.14), NS - Burden laboratory tests: mean 1.87 (SD 1.31) versus 1.04 (SD 1.17), *p* < 0.001 After correction for baseline differences, QoL in the S-OPAT group was significantly lower than in the oral antibiotic group: difference 8.3 points, *p* < 0.001
Saillen, 2017	Adult patients	71	Survey	- S-OPAT patients scored significantly better (after adjustment for age, sex and treatment duration) on 6/17 questions, compared to OPAT patients - Overall satisfaction was statistically higher in S-OPAT patients compared to OPAT patients: 96% versus 80% (OR 5.0, 95%CI 1.19-20.0)
Descriptive studies				
Bodycot, 2020	Adult patients	105	Self-constructed questionnaire	Preference for AB treatment at home: 29/32 (90.6%)
Eaves, 2014	Adult patients and caregivers	Patients 29 Caregivers 9	Reassessment of competence by specialist nurse. Median interval between completion of initial training and reassessment: 35 d. (range 12–118)	- Fully competent: 35/38 (92%) - Patient made a mistake with immediate correction: 2/38 (5%) - Patient made a mistake and continued with the procedure: 1/38 (3%)
Keller, 2022	Adult patients	20	Survey at least 2 weeks after discharge	- Comfortable infusing medication at discharge: 18/20 (90%) - Comfortable infusing medication 2 weeks after discharge: 20/20 (100%) - Comfortable with bathing with the IV catheter: 16/20 (80%) - Satisfaction with the training: 19/20 (95%) - Satisfaction with managing the IV catheter at home: 20/20 (100%)
Kieran, 2009	Adult patients	12	Standardized telephone survey	- Preference for AB treatment at home: 12/12 (100%) - Satisfaction with the service and the instructions: 12/12 (100%)
Kilinc, 2023 (a)	Adult patients	52	Self-constructed survey	- Satisfaction with S-OPAT: 51/52 (98.1%) - No problem managing S-OPAT at home: 43/52 (82.7%) - Ability to carry out tasks at home: 35/52 (67.3%) - Ability to return to work: 16/52 (30.8%) - Self-perceived good health: 48/52 (92.3%)
Kilinc, 2023 (b)	Adult patients	33	Self-constructed survey	- Satisfaction with S-OPAT: 28/33 (84.8%) - Ability to carry out tasks at home: 19/33 (57.6%)
Lopez-Cortes, 2019	Adult patients	67	Survey	- Preference for AB treatment at home: 66/67 (99%) - Significant improvement in quality of life: 39/45 (86.7%)
Yagnik, 2022	Adult patients	Competency 200 Survey 22	Competency assessment by teach-back method Satisfaction survey	- Competency: 82% achieved acceptable level in ≤ 3 attemps - Satisfaction survey: 96% of participating patients chose the syringe method over the IV drip
Qualitative studies				
Keller, 2019	Adult patients	Interviews 29 Observations 14	Semi-structured interviews. Contextual inquiry tools for the observations	Hazards related to physical attributes in patients’ home environments: - Unclear how to bath with catheter - Pets shed fur, create waste and tug on catheter - Extremes in temperature (warm and cold) - Household clutter, e.g. patients cannot access certain parts of the home - Indoor soil and food, e.g. physical state makes it difficult to perform household tasks - Outdoor work: exposed to soil or dirt - Travel, e.g. patient must carry supplies and perform tasks outside of the home
Keller, 2020 (a)	Adult patients and caregivers	Patients 7 Caregivers 3	Focus group discussions	Barriers most frequently mentioned: - Healthcare professionals may not always with each other about the patient’s care - Instruction is rushed - Uncertainty how to bath with a catheter - Patients and caregivers must devote a lot of time for S-OPAT tasks - The catheter gets caught as the patient moves around the house
Keller, 2020 (b)	Adult patients	Interviews 40 Observations 20	Semi-structured interviews Contextual inquiry tools for the observations	- Patients appreciate visual cognitive aids to remember (sub)tasks - Patients struggle to understand instructions - Patients are uncertain about several instructions, e.g. hand washing, the temperature of the medication - Patients struggle to detect incidents, and do not always respond to alerts
Twiddy, 2018	Adult patients	Patients 5	Semi-structured interviews	- Multiple treatments each day left the patients with little time to fit anything else into the day - Maintaining aseptic technique, correct storage of medication and administering the drugs became routine for patients, but all were aware of the consequences of any lapse of judgement. Reminders by nurses were appreciated - Formal training and ongoing support by nurses were considered essential and enabled them to be fully involved in decisions about their care

## Discussion

This scoping review aimed to give a comprehensive overview of publications reporting on the clinical outcomes and safety of S-OPAT, factors impacting these outcomes, and experiences of patients and caregivers with S-OPAT. After systematically searching the literature of the past 17 years, a total of 44 studies were included in this review. Clinical outcomes were reported in 17 and safety in 23 publications. Many publications were descriptive and reported on the evaluation of S-OPAT care in a single cohort. Eleven studies compared S-OPAT to other OPAT delivery models. These studies showed that clinical outcomes were generally comparable. Two studies found increased adverse events with S-OPAT, while the majority reported no safety concerns. Seven potential risk factors were explored in 9 studies: age, drug use, comorbidity, microbiological diagnosis, antimicrobial agent, catheter type, and delivery method. Risk factors for adverse events included older age, comorbidities and *staphylococcus aureus* infection. Fourteen studies reported on patient-centred outcomes and showed that patients and caregivers considered S-OPAT a suitable alternative for other OPAT delivery models.

Healthcare systems will face substantial challenges in the coming decades. The aging population and the expected increase in chronic and acute diseases will result in a growing care demand. To ensure the availability, quality and affordability of care, efforts will need to be made to reduce unnecessary hospital stays and to increase the reliance on informal care. It is expected that patients will become increasingly dependent on family support [53]. This development also affects OPAT care, highlighting the urgent need for the implementation of an S-OPAT pathway. The studies in our review suggest that S-OPAT is an acceptable healthcare model for suitable patients. As safety is the main concern, healthcare professionals should anticipate preparing patients and family members during admission for the period after discharge to guarantee safety and optimal clinical outcome [54].

We identified a substantial degree of heterogeneity in the included studies. We observed substantial variation in patient populations, infectious disease diagnoses, and treatment characteristics (e.g., choice of antimicrobial agents or catheter type). Also, the varying comparators used in the comparative studies of S-OPAT makes it difficult to draw overarching conclusions. For example, OPAT care might be delivered by a community nurse at the patients’ homes, or within a specialized clinic or infusion centre, where patients attend an OPAT facility daily [55].

Moreover, the definitions of clinical outcome and safety differed across studies. For example, clinical outcome was reported as treatment success, cure, improvement or deterioration, and safety was recorded as drug-related adverse events, catheter complications, readmissions or mortality. These inconsistencies in outcome reporting make it impossible to make meaningful comparisons between studies, thereby hindering the ability to reach firm conclusions as previously noted [8, 56, 57]. To overcome this problem, the development of a core outcome set (COS) for research on OPAT delivery models might contribute to more consistency, making it easier for study results to be compared and combined as appropriate [58], and implemented [59]. This scoping review could be the first step, but obviously more work needs to be done, i.e. patient and caregiver consultation to identify the outcomes they consider relevant, and a Delphi study to reach consensus among experts. The previously developed set of quality indicators to assess and improve the quality of OPAT care may be helpful to move forward in this field [60].

Careful patient selection is considered crucial to guarantee a successful S-OPAT clinical pathway [1]. Selection criteria for OPAT are well described [61], but additional criteria are needed for S-OPAT. As self-administration is the key difference compared to OPAT delivery models, the additional criteria should focus on training and self-management skills, compliance of patients, the role of caregivers, and the safety and suitability of the home environment. The included studies in this review describing risk factors for adverse events during S-OPAT, did not describe these factors specifically related to S-OPAT.

Previous studies have found positive associations between patients’ and caregivers’ experiences and clinical outcome and safety among a wide range of health conditions [62, 63]. However, we found that only few of the clinical studies included in this review described patient-centred outcomes of S-OPAT. The experiences of caregivers were explored even less. Furthermore, constructs explored varied (e.g., satisfaction, comfort, experiences, self-efficacy) and were mainly measured with self-constructed questionnaires. As one of the main reasons to initiated S-OPAT is to improve the quality of life for both patients and caregivers [2], this outcome measure deserves additional attention. Validated tools are available, such as EQ-5D-5 L [64] and CarerQoL-7D [65]. Also, confidence in managing health issues, and experienced burden are considered important outcome measures with the increasing involvement of patients and families in healthcare [62]. Again, these outcomes can be assessed using validated tools, such as the Health Confidence Score (HCS) [66], and the Caregiver Strain Index plus (CSI+) [67]. In addition, qualitative studies are valuable to obtain more in-depth information. Measuring patient reported outcome measures should be integrated in the S-OPAT pathway to provide optimal support for both patients and caregivers [68].

This scoping review has strengths and limitations. Firstly, we followed the preferred methodology of executing and reporting a scoping review and retrieved relevant articles using 4 distinct databases: MEDLINE, CINAHL, Embase and Cochrane library. Secondly, we studied nearly all studies full text as OPAT and S-OPAT are terms often used interchangeable. However, we limited the search to the past 17 years which may have resulted in missing publications published before that timeframe. However, older publications are less representative of the current state of S-OPAT literature, which we aimed to describe. In addition, we did not report on the level of evidence of the included studies. Performing critical appraisal was beyond the aim of this scoping review and is not required in the JBI methodology for scoping reviews. This means that this review provides a description of relevant publications to inform clinical practice on S-OPAT and does not attempt to draw conclusions based on their results.

Besides the above-mentioned directions for future research, the role of artificial intelligence should be explored as an innovative approach to predict clinical outcome and safety for OPAT patients in different delivery models [69]. Another important research topic is the ecological footprint that comes with antimicrobial treatment and the different delivery models. It is widely recognized that healthcare has a large climate impact [70].

Based on the gaps of knowledge, we started research projects to investigate factors that contribute to successful S-OPAT care, experiences of patients and their caregivers, as well as the ecological footprint of different OPAT models.

## Conclusion

This scoping review identified studies on S-OPAT with considerable variation in outcome measures, and a notable lack of research on patients and caregivers experiences. The growing care demand now and in the future urges further development of S-OPAT care. Recommendations for future research are formulated. Attention should be paid to robust study methodology and reporting, promoting homogeneity in outcome measures.

## Data Availability

No datasets were generated or analysed during the current study.
